# Supplementary material for: Pet-Human Gut Microbiome Host Classifier Using Data from Different Studies
Source: Microorganisms. 2020 Oct 15;8(10):1591. doi: 10.3390/microorganisms8101591 (PMC7602744; doi:10.3390/microorganisms8101591)
Supplement: Supplementary file 1 [file microorganisms-08-01591-s001.zip › supplements/FigureS3.pdf]

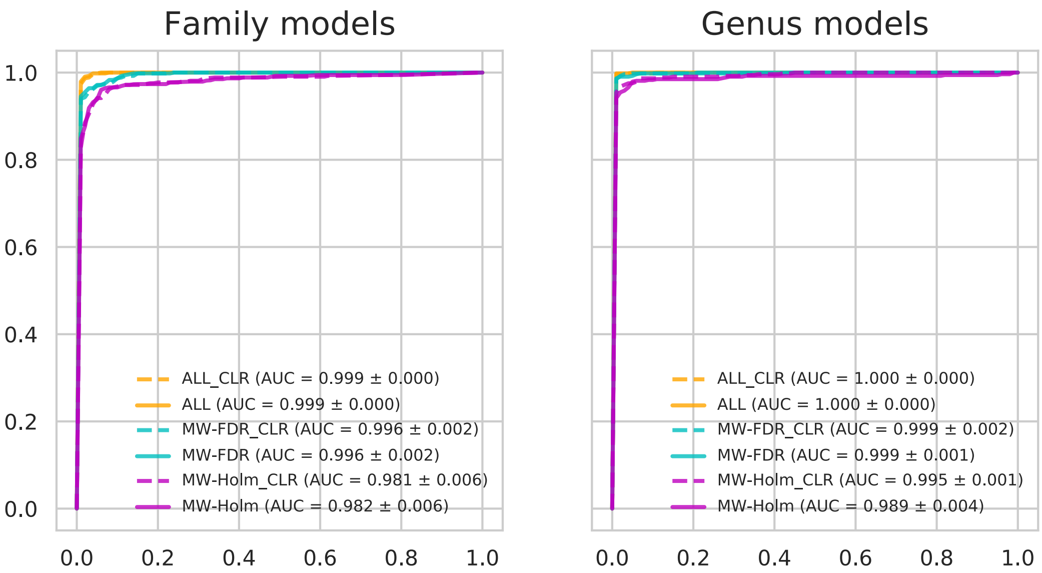

Supplementary Figure 3. ROC-curves for family (A) and genus (B) models obtained in cross-validation (cv=5). Full models are shown in orange, models restricted by features selected by MW-test using FDR correction are in blue, models restricted by features selected by MW-test using Holm correction are in magenta. Models that used CLR-transformed data as an input are shown with dashed lines.
