# Supplementary material for: Pet-Human Gut Microbiome Host Classifier Using Data from Different Studies
Source: Microorganisms. 2020 Oct 15;8(10):1591. doi: 10.3390/microorganisms8101591 (PMC7602744; doi:10.3390/microorganisms8101591)
Supplement: Supplementary file 1 [file microorganisms-08-01591-s001.zip › supplements/FigureS1.pdf]

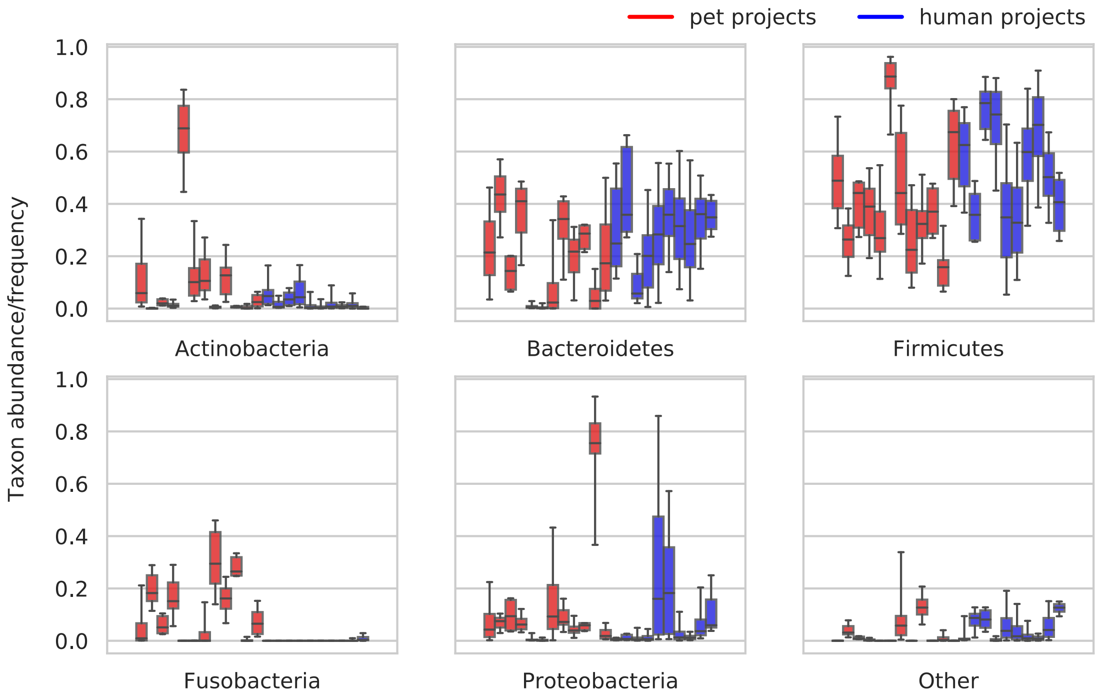

Supplementary Figure 1. The boxplot of the phylum abundance for the projects used in the study. The pet projects are shown in red, human projects are in blue.
