# Supplementary material for: Pet-Human Gut Microbiome Host Classifier Using Data from Different Studies
Source: Microorganisms. 2020 Oct 15;8(10):1591. doi: 10.3390/microorganisms8101591 (PMC7602744; doi:10.3390/microorganisms8101591)
Supplement: Supplementary file 1 [file microorganisms-08-01591-s001.zip › supplements/FigureS4.pdf]

A

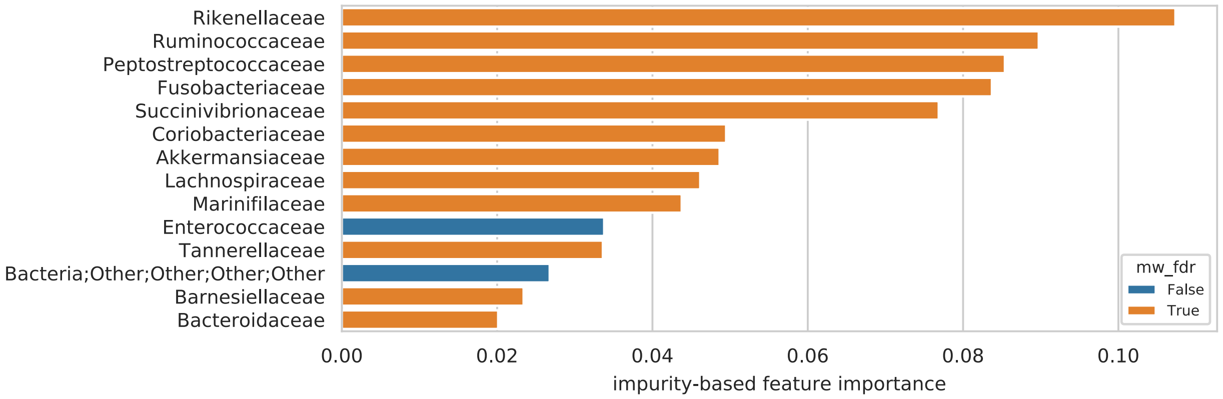

B

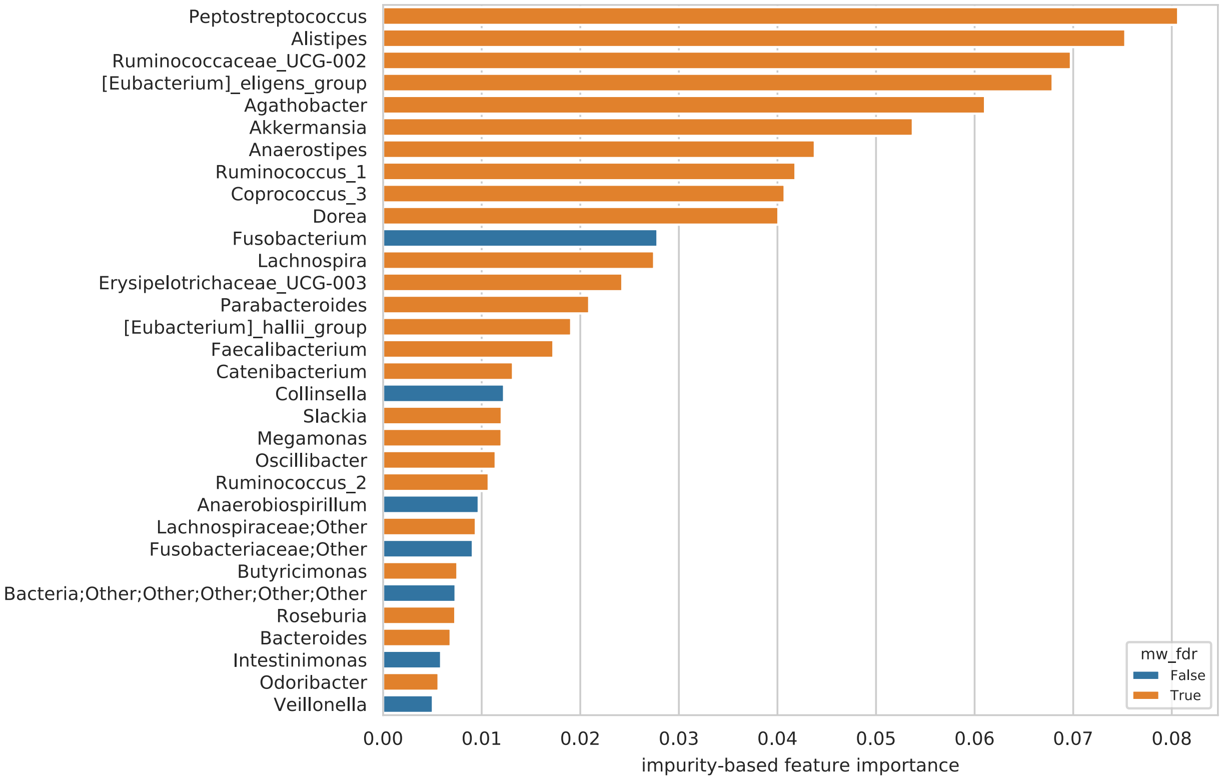

Supplementary Figure 4. Top families (A) and genera (B) important for human/pet host discrimination as suggested by Family-ALL and Genus-ALL models. The taxa also suggested by MW test are shown in orange. At the family level, the top 14 taxa are shown, at the genus level top 32 taxa are shown; this corresponds to the numbers of taxa selected by the MW test with FDR correction.
