# Supplementary material for: Pet-Human Gut Microbiome Host Classifier Using Data from Different Studies
Source: Microorganisms. 2020 Oct 15;8(10):1591. doi: 10.3390/microorganisms8101591 (PMC7602744; doi:10.3390/microorganisms8101591)
Supplement: Supplementary file 1 [file microorganisms-08-01591-s001.zip › supplements/FigureS5.pdf]

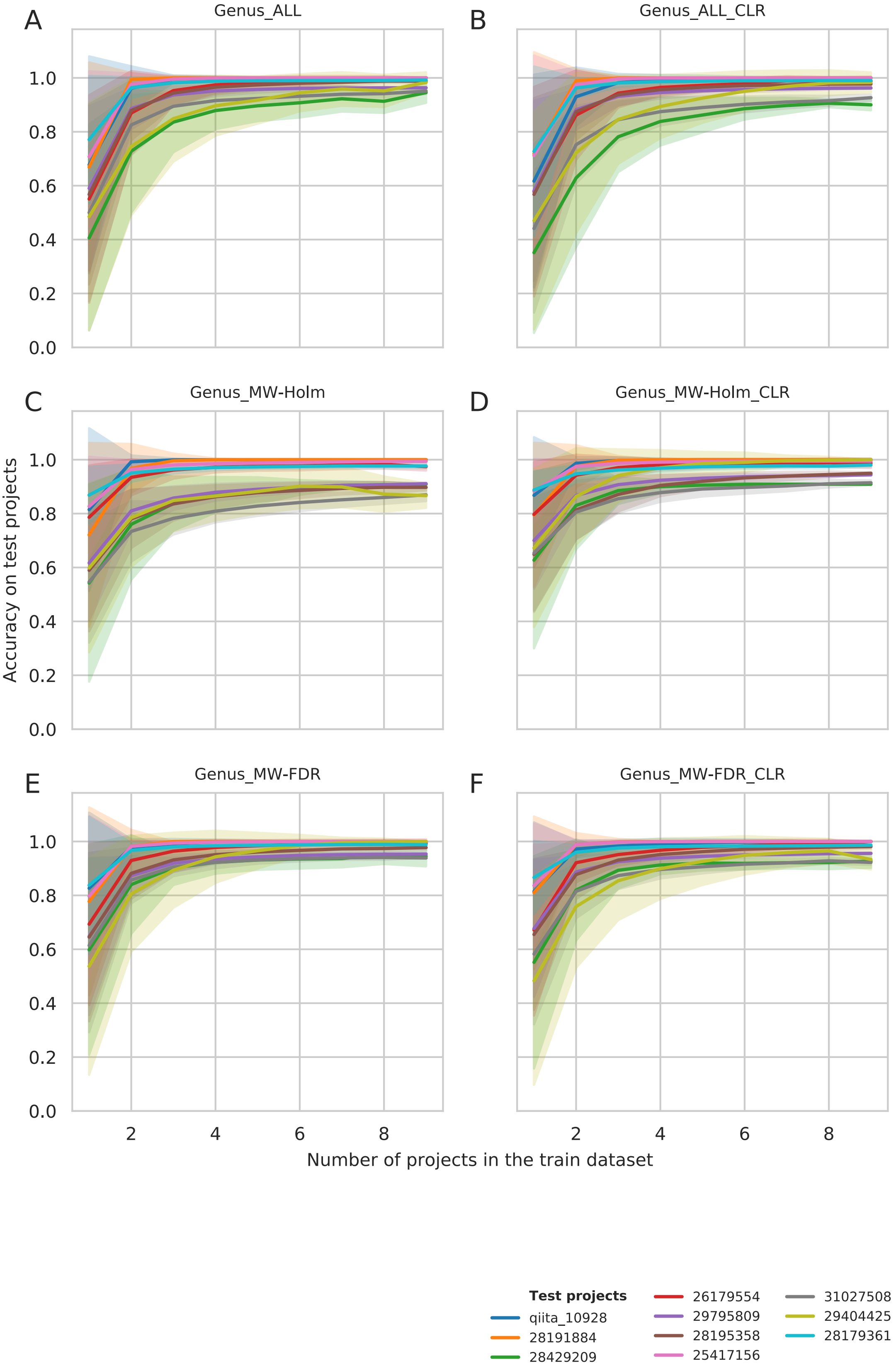

Supplementary Figure 5. The dependency of accuracy on test human projects from the number of human projects used in the training set (for the genus models). The average value of accuracy among all the models (for the specific *n*) for each test project is shown, see in-plot legend for specific color-project mapping.
