# Supplementary material for: Pet-Human Gut Microbiome Host Classifier Using Data from Different Studies
Source: Microorganisms. 2020 Oct 15;8(10):1591. doi: 10.3390/microorganisms8101591 (PMC7602744; doi:10.3390/microorganisms8101591)
Supplement: Supplementary file 1 [file microorganisms-08-01591-s001.zip › supplements/TextS1.pdf]

# Pet-human gut microbiome host classifier using data from different studies

**Nadia Bykova[1,*], Nikita Litovka[1], Anna Popenko[1] & Sergey Musienko[1]**

[1] Atlas LLC, Malaya Nikitskaya 31, Moscow 121069, Russia
* Correspondence: bykova@atlasbiomed.com

## Supplementary Text 1

The following data describe the process of parameters selection of random forest models. The models parameters were selected in two steps. First, best combination of parameters is selected by the best accuracy achieved in the cross-validation experiment (the number of folds is 5). The parameters ranges used at this first step were:

- 'n_estimators': {100},
- 'max_depth': [2,12],
- 'min_samples_split': [2,10],
- 'max_features': [1,sqrt(features #)].

At the second step, each parameter was varied in a wider range while all the other were fixed at their best values defined at the first step. The ranges for parameters at the second step were:

- 'n_estimators': {1,5,10,50,100,500,1000},
- 'max_depth': [2,30],
- 'min_samples_split': [2,30],
- 'max_features': [1, features #].

Note that even for a few models best 'n_estimators' parameter was less than 100, we still restrcted it with minimun 100 trees. The best parameters obtained at the second step were used to train the model at the whole dataset. Out-of-bag (OOB) esetimations of model performance were reported.

Below the following data is present for each model:

1. Dependency of CV accuracy on the value of each parameter at the second step,
2. The best parameters obtained at first and second steps,
3. ROC curve for the model with best parameters estimated from the CV results,
4. The OOB-scores of the final model.

Model: Family_ALL_CLR

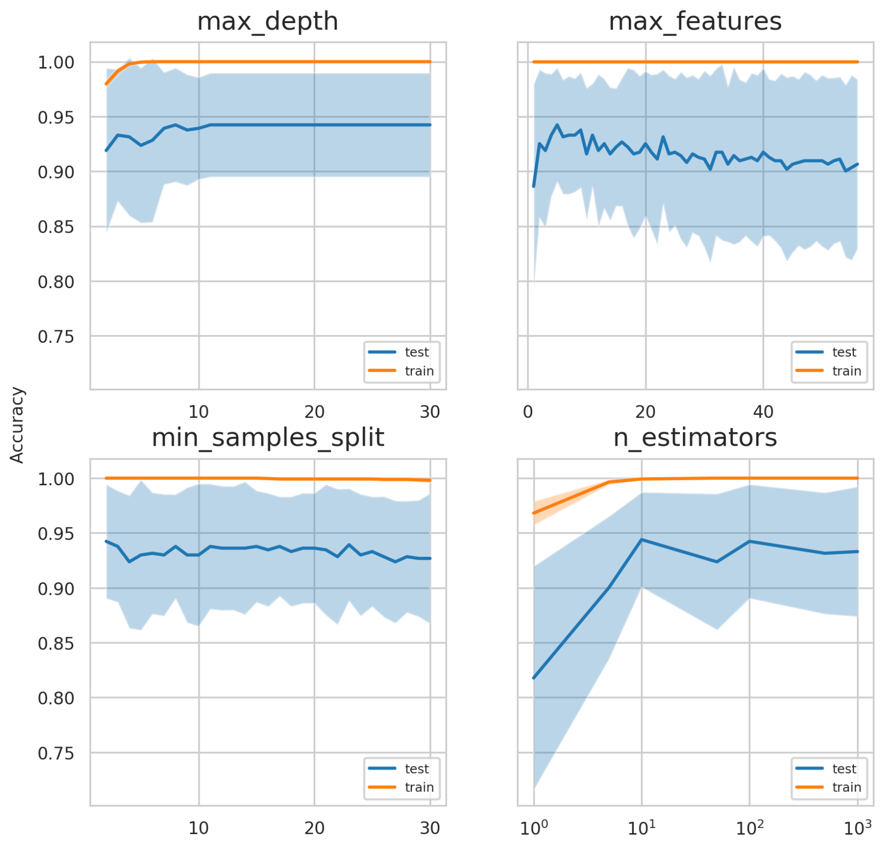


Figure 1. Parameters selection (step 2): Dependancy of accuracy at test (blue) and train (orange) parts of the dataset on the parameter value is shown. The mean+/-standard deviation of 5 folds is shown.

Best parameters 1:{'max_depth': 8, 'max_features': 5, 'min_samples_split': 2, 'n_estimators': 100}

Best parameters 2:{'max_depth': 8, 'max_features': 5, 'min_samples_split': 2, 'n_estimators': 100}

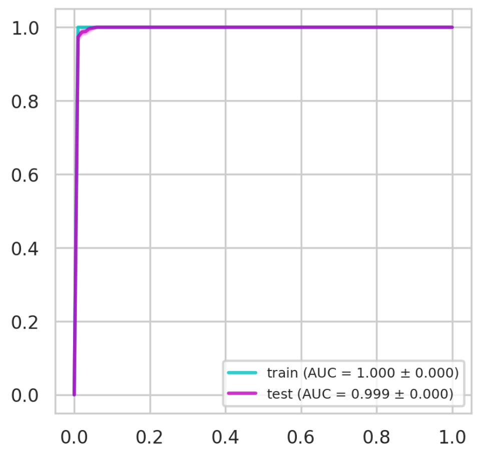


Figure 2. ROC curve at test (magenta) and train (blue) parts of the dataset is shown. The mean+/-standard deviation of 5 folds is shown.

OOB estimations: {'oob_accuracy': '0.981 ± 0.004', 'oob_f1_score': '0.980 ± 0.004', 'oob_precision': '0.987 ± 0.004', 'oob_recall': '0.974 ± 0.006'}

# Model: Family_ALL

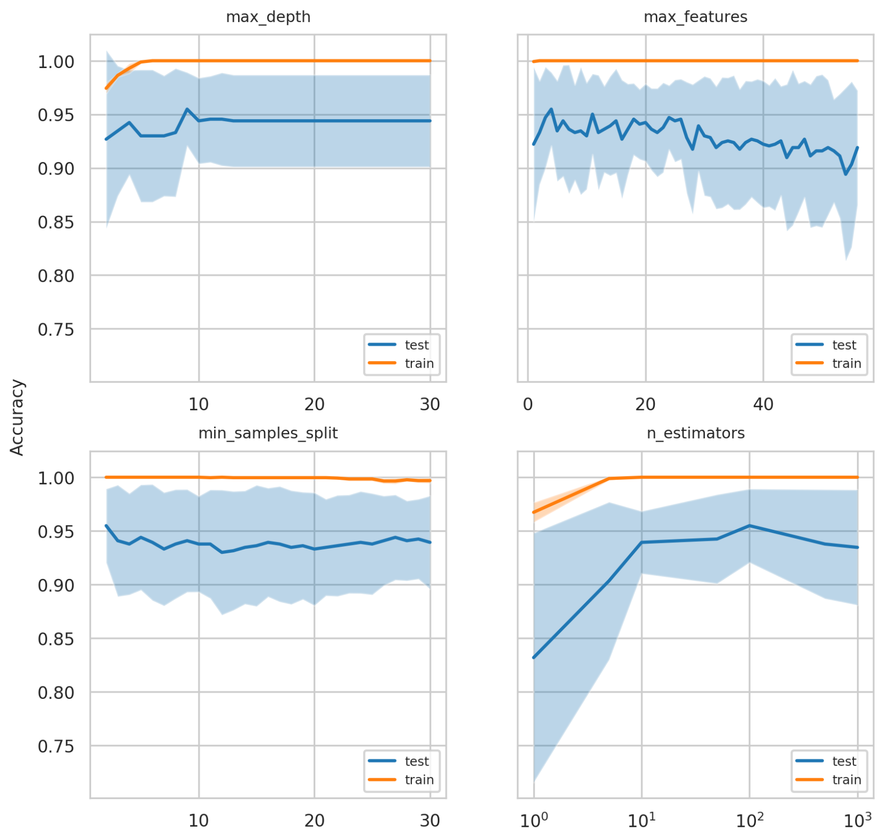


Figure 1. Parameters selection (step 2): Dependancy of accuracy at test (blue) and train (orange) parts of the dataset on the parameter value is shown. The mean+/-standard deviation of 5 folds is shown.

Best parameters 1:{'max_depth': 9, 'max_features': 4, 'min_samples_split': 2, 'n_estimators': 100}

Best parameters 2:{'max_depth': 9, 'max_features': 4, 'min_samples_split': 2, 'n_estimators': 100}

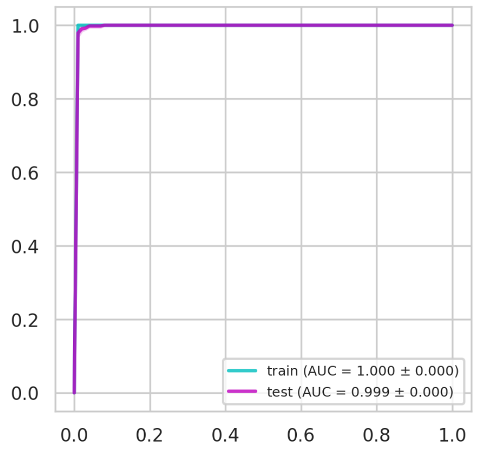


Figure 2. ROC curve at test (magenta) and train (blue) parts of the dataset is shown. The mean+/-standard deviation of 5 folds is shown.

OOB estimations: {'oob_accuracy': '0.983 ± 0.004', 'oob_f1_score': '0.983 ± 0.004', 'oob_precision': '0.989 ± 0.004', 'oob_recall': '0.977 ± 0.006'}

## Model: Family_MW-FDR_CLR

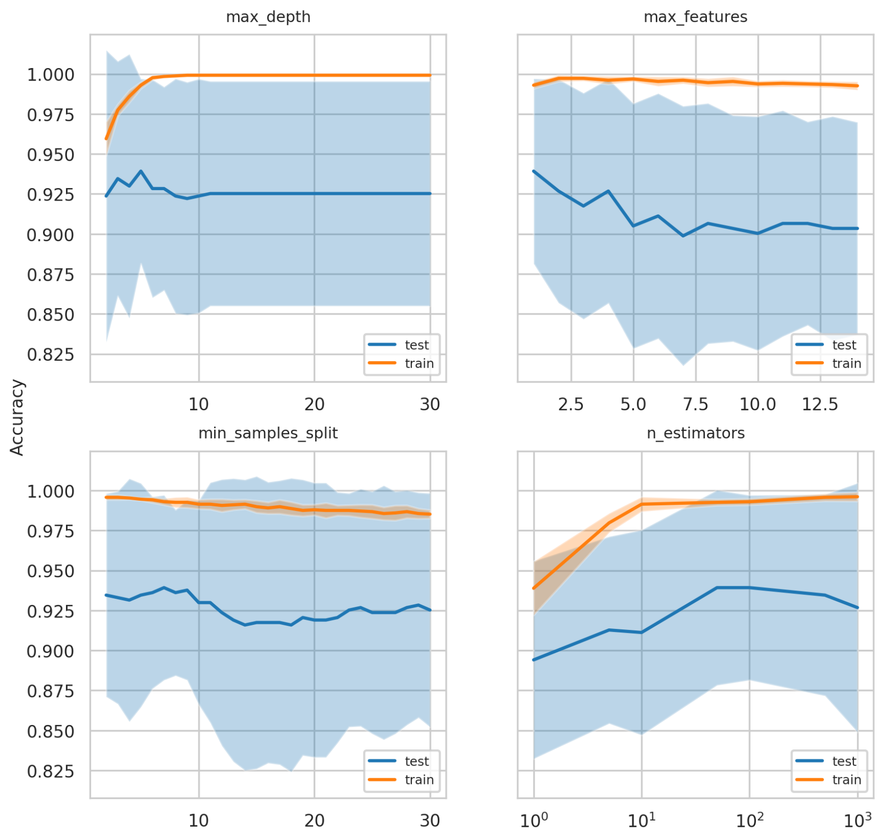


Figure 1. Parameters selection (step 2): Dependancy of accuracy at test (blue) and train (orange) parts of the dataset on the parameter value is shown. The mean+/-standard deviation of 5 folds is shown.

Best parameters 1:{'max_depth': 5, 'max_features': 1, 'min_samples_split': 7, 'n_estimators': 100}

Best parameters 2:{'max_depth': 5, 'max_features': 1, 'min_samples_split': 7, 'n_estimators': 100}

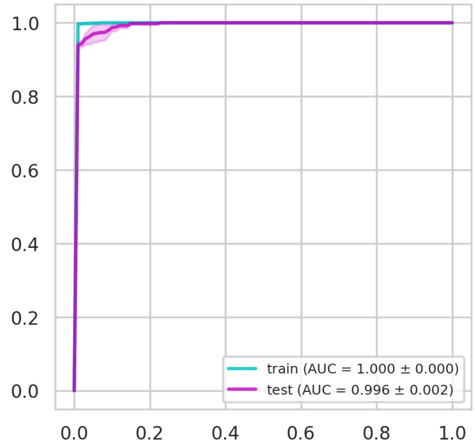


Figure 2. ROC curve at test (magenta) and train (blue) parts of the dataset is shown. The mean+/-standard deviation of 5 folds is shown.

OOB estimations: {'oob_accuracy': '0.966 ± 0.004', 'oob_f1_score': '0.966 ± 0.004', 'oob_precision': '0.976 ± 0.005', 'oob_recall': '0.955 ± 0.006'}

Model: Family_MW-FDR

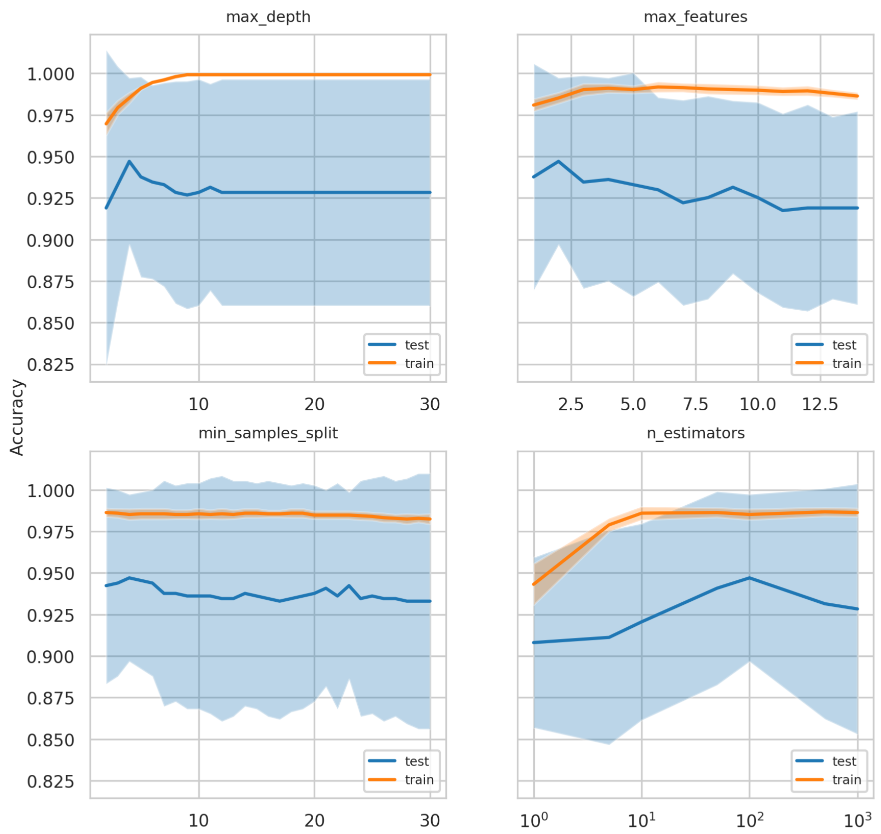


Figure 1. Parameters selection (step 2): Dependancy of accuracy at test (blue) and train (orange) parts of the dataset on the parameter value is shown. The mean+/-standard deviation of 5 folds is shown.

Best parameters 1:{'max_depth': 4, 'max_features': 2, 'min_samples_split': 4, 'n_estimators': 100}

Best parameters 2:{'max_depth': 4, 'max_features': 2, 'min_samples_split': 4, 'n_estimators': 100}

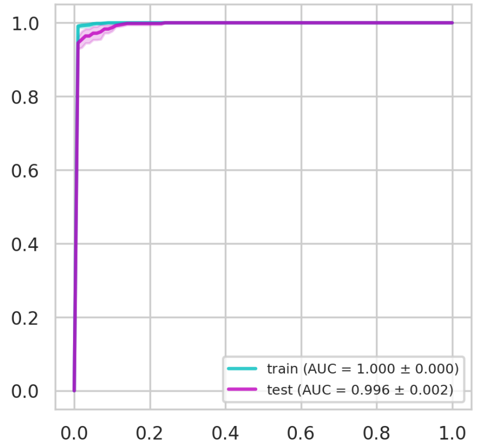


Figure 2. ROC curve at test (magenta) and train (blue) parts of the dataset is shown. The mean+/-standard deviation of 5 folds is shown.

OOB estimations: {'oob_accuracy': '0.970 ± 0.003', 'oob_f1_score': '0.970 ± 0.003', 'oob_precision': '0.986 ± 0.004', 'oob_recall': '0.955 ± 0.005'}

Model: Family_MW-Holm_CLR

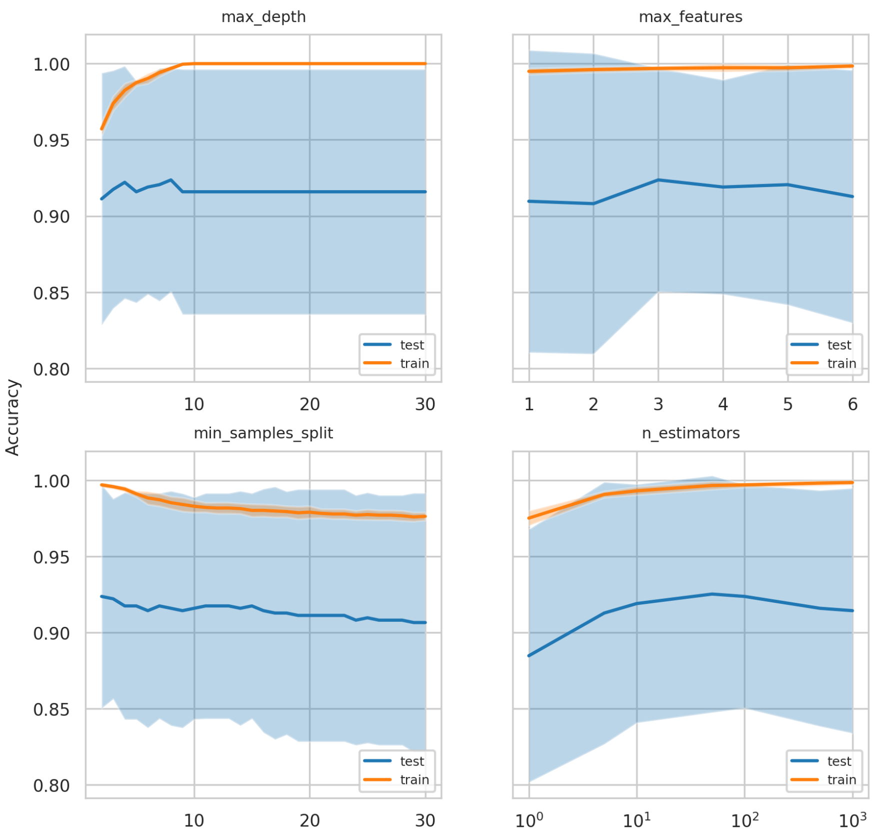


Figure 1. Parameters selection (step 2): Dependancy of accuracy at test (blue) and train (orange) parts of the dataset on the parameter value is shown. The mean+/-standard deviation of 5 folds is shown.

Best parameters 1:{'max_depth': 8, 'max_features': 3, 'min_samples_split': 2, 'n_estimators': 100}

Best parameters 2:{'max_depth': 8, 'max_features': 3, 'min_samples_split': 2, 'n_estimators': 100}

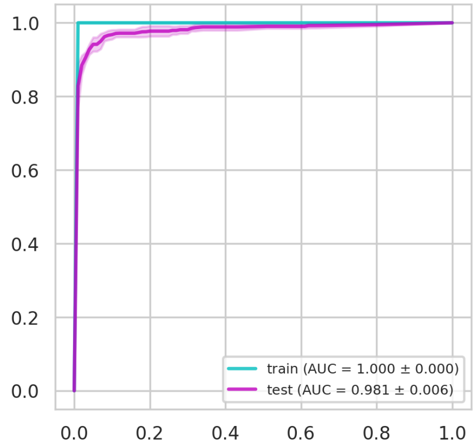


Figure 2. ROC curve at test (magenta) and train (blue) parts of the dataset is shown. The mean+/-standard deviation of 5 folds is shown.

OOB estimations: {'oob_accuracy': '0.954 ± 0.003', 'oob_f1_score': '0.954 ± 0.003', 'oob_precision': '0.957 ± 0.004', 'oob_recall': '0.951 ± 0.004'}

Model: Family_MW-Holm

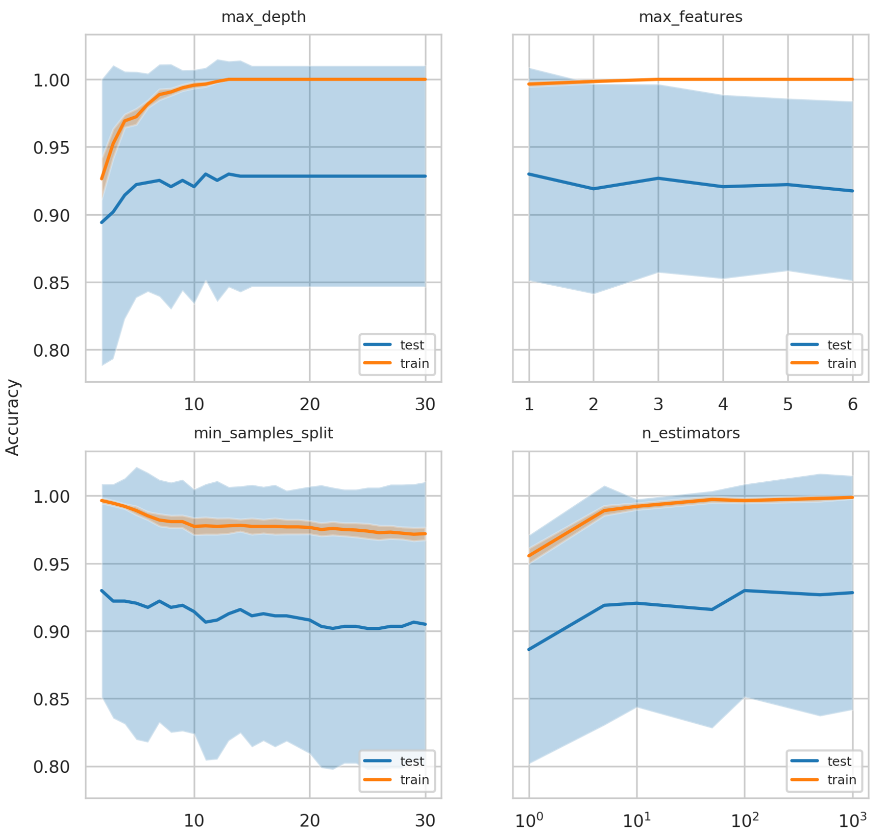


Figure 1. Parameters selection (step 2): Dependancy of accuracy at test (blue) and train (orange) parts of the dataset on the parameter value is shown. The mean+/-standard deviation of 5 folds is shown.

Best parameters 1:{'max_depth': 11, 'max_features': 1, 'min_samples_split': 2, 'n_estimators': 100}

Best parameters 2:{'max_depth': 11, 'max_features': 1, 'min_samples_split': 2, 'n_estimators': 100}

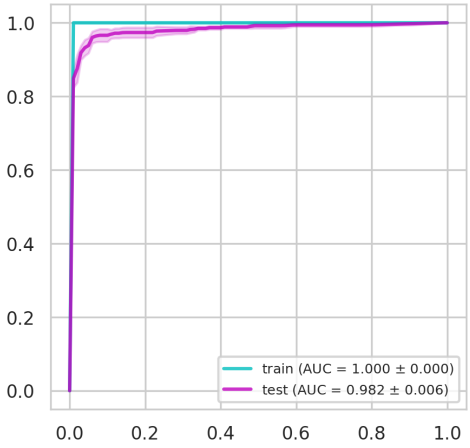


Figure 2. ROC curve at test (magenta) and train (blue) parts of the dataset is shown. The mean+/-standard deviation of 5 folds is shown.

OOB estimations: {'oob_accuracy': '0.953 ± 0.004', 'oob_f1_score': '0.953 ± 0.003', 'oob_precision': '0.951 ± 0.006', 'oob_recall': '0.955 ± 0.004'}

Model: Genus_ALL_CLR

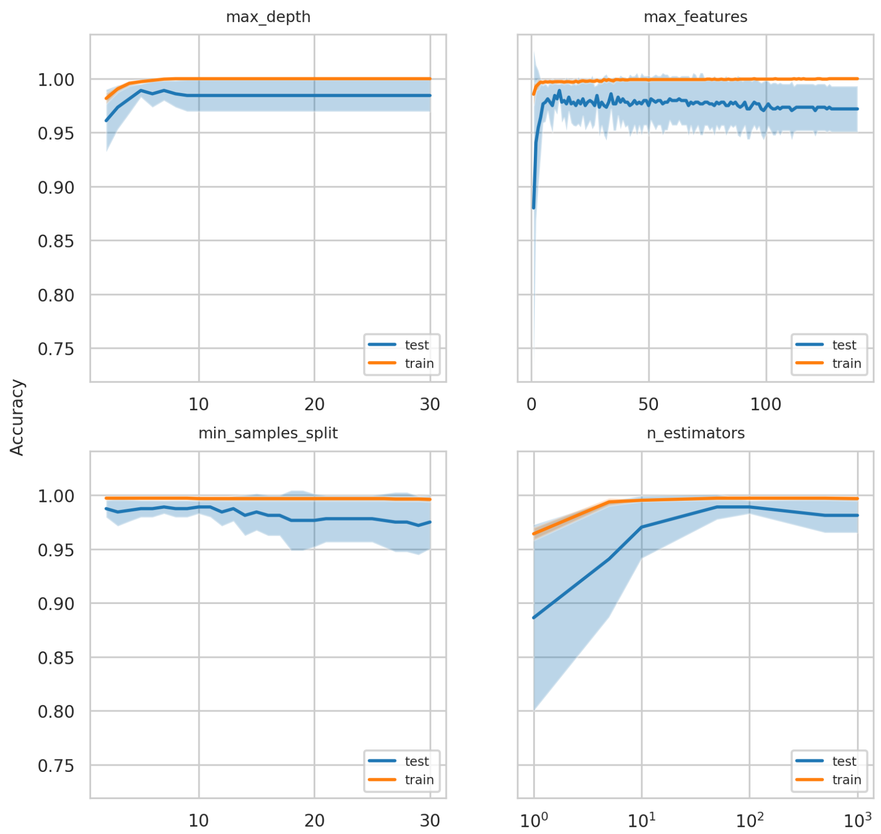


Figure 1. Parameters selection (step 2): Dependancy of accuracy at test (blue) and train (orange) parts of the dataset on the parameter value is shown. The mean+/-standard deviation of 5 folds is shown.

Best parameters 1:{'max_depth': 5, 'max_features': 12, 'min_samples_split': 7, 'n_estimators': 100}

Best parameters 2:{'max_depth': 5, 'max_features': 12, 'min_samples_split': 7, 'n_estimators': 100}

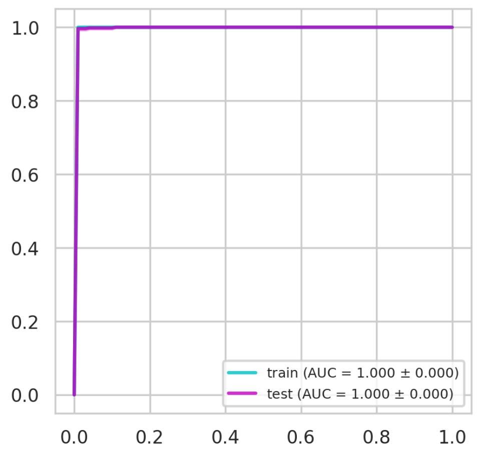


Figure 2. ROC curve at test (magenta) and train (blue) parts of the dataset is shown. The mean+/-standard deviation of 5 folds is shown.

OOB estimations: {'oob_accuracy': '0.990 ± 0.002', 'oob_f1_score': '0.990 ± 0.002', 'oob_precision': '0.999 ± 0.001', 'oob_recall': '0.981 ± 0.003'}

# Model: Genus_ALL

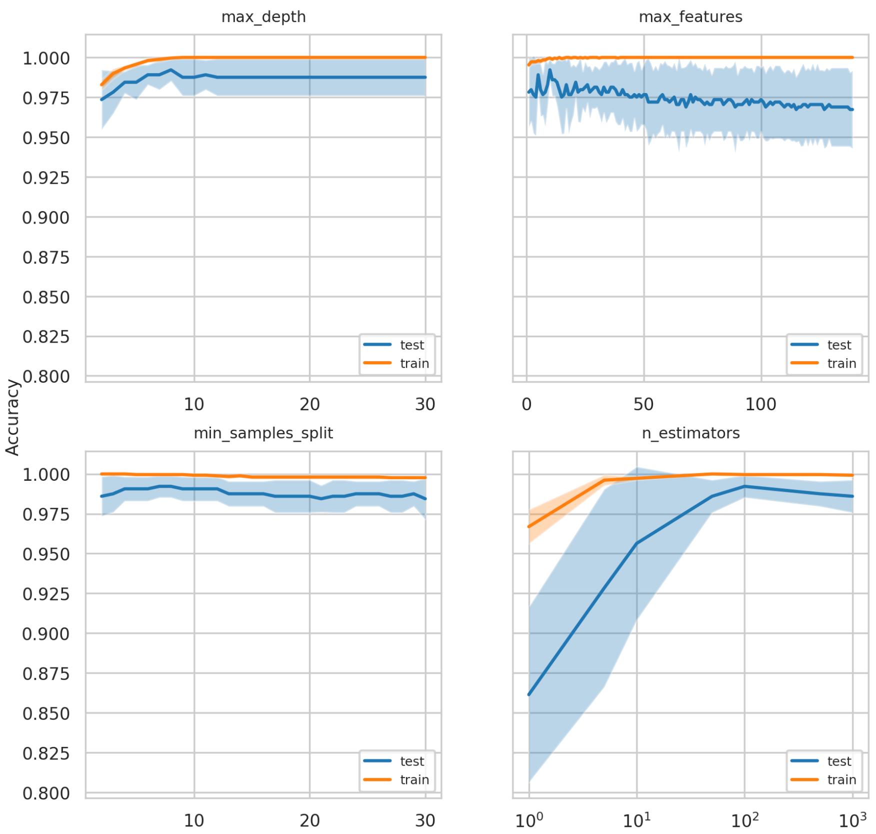


Figure 1. Parameters selection (step 2): Dependancy of accuracy at test (blue) and train (orange) parts of the dataset on the parameter value is shown. The mean+/-standard deviation of 5 folds is shown.

Best parameters 1:{'max_depth': 8, 'max_features': 10, 'min_samples_split': 7, 'n_estimators': 100}

Best parameters 2:{'max_depth': 8, 'max_features': 10, 'min_samples_split': 7, 'n_estimators': 100}

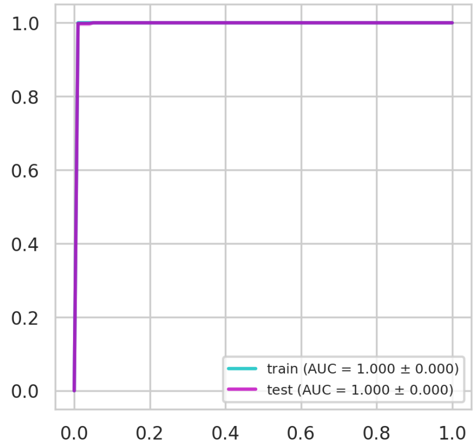


Figure 2. ROC curve at test (magenta) and train (blue) parts of the dataset is shown. The mean+/-standard deviation of 5 folds is shown.

OOB estimations: {'oob_accuracy': '0.992 ± 0.002', 'oob_f1_score': '0.992 ± 0.002', 'oob_precision': '0.999 ± 0.002', 'oob_recall': '0.985 ± 0.003'}

Model: Genus_MW-FDR_CLR

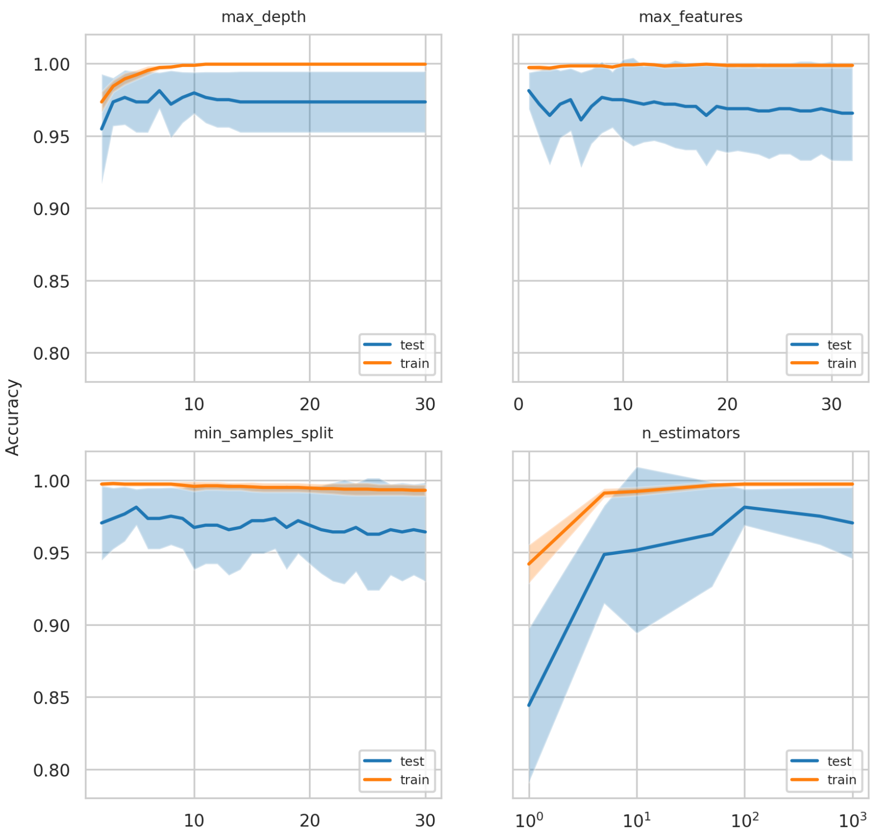


Figure 1. Parameters selection (step 2): Dependancy of accuracy at test (blue) and train (orange) parts of the dataset on the parameter value is shown. The mean+/-standard deviation of 5 folds is shown.

Best parameters 1:{'max_depth': 7, 'max_features': 1, 'min_samples_split': 5, 'n_estimators': 100}

Best parameters 2:{'max_depth': 7, 'max_features': 1, 'min_samples_split': 5, 'n_estimators': 100}

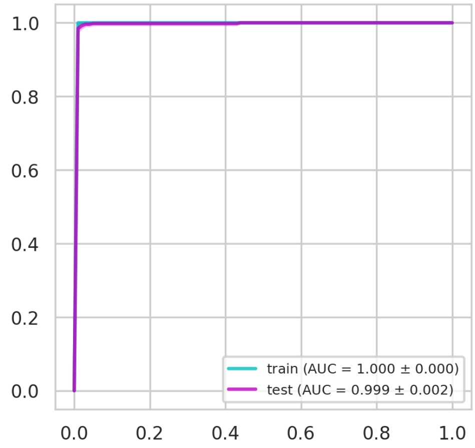


Figure 2. ROC curve at test (magenta) and train (blue) parts of the dataset is shown. The mean+/-standard deviation of 5 folds is shown.

OOB estimations: {'oob_accuracy': '0.986 ± 0.002', 'oob_f1_score': '0.985 ± 0.002', 'oob_precision': '0.997 ± 0.003', 'oob_recall': '0.974 ± 0.004'}

Model: Genus_MW-FDR

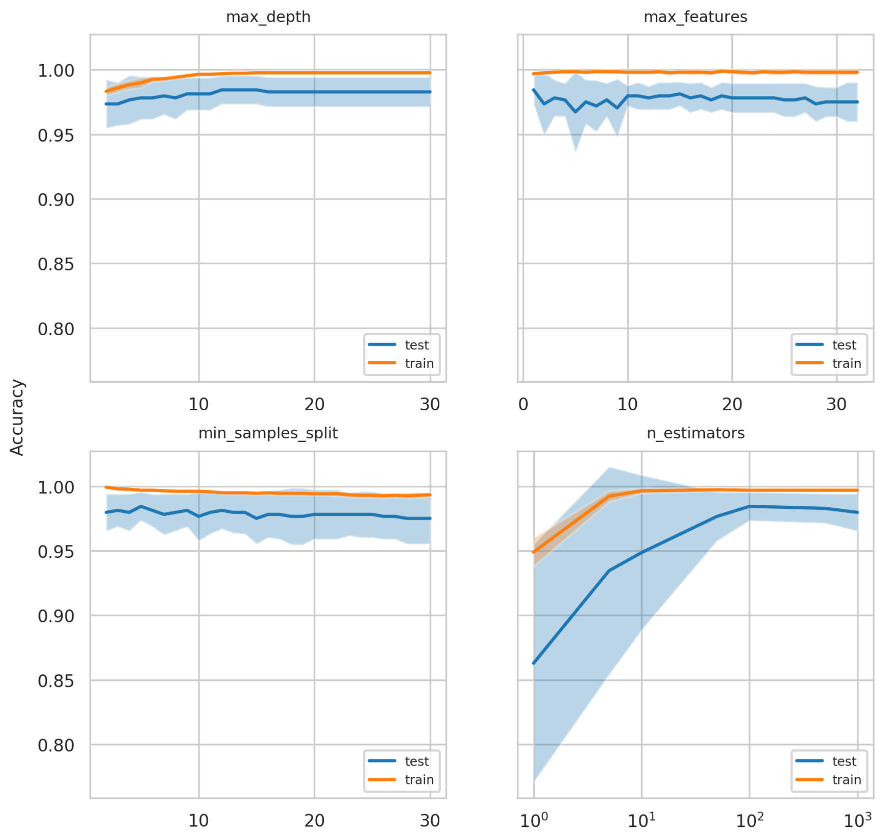


Figure 1. Parameters selection (step 2): Dependancy of accuracy at test (blue) and train (orange) parts of the dataset on the parameter value is shown. The mean+/-standard deviation of 5 folds is shown.

Best parameters 1:{'max_depth': 12, 'max_features': 1, 'min_samples_split': 5, 'n_estimators': 100}

Best parameters 2:{'max_depth': 12, 'max_features': 1, 'min_samples_split': 5, 'n_estimators': 100}

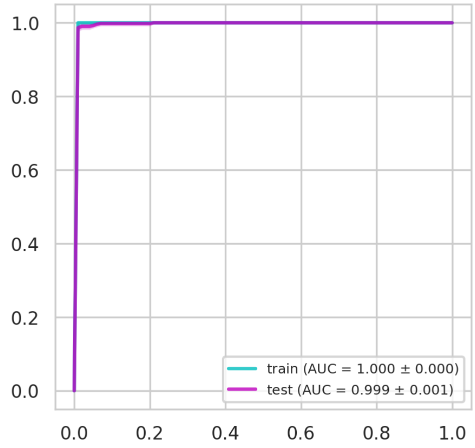


Figure 2. ROC curve at test (magenta) and train (blue) parts of the dataset is shown. The mean+/-standard deviation of 5 folds is shown.

OOB estimations: {'oob_accuracy': '0.989 ± 0.002', 'oob_f1_score': '0.989 ± 0.002', 'oob_precision': '0.998 ± 0.003', 'oob_recall': '0.979 ± 0.003'}

Model: Genus_MW-Holm_CLR

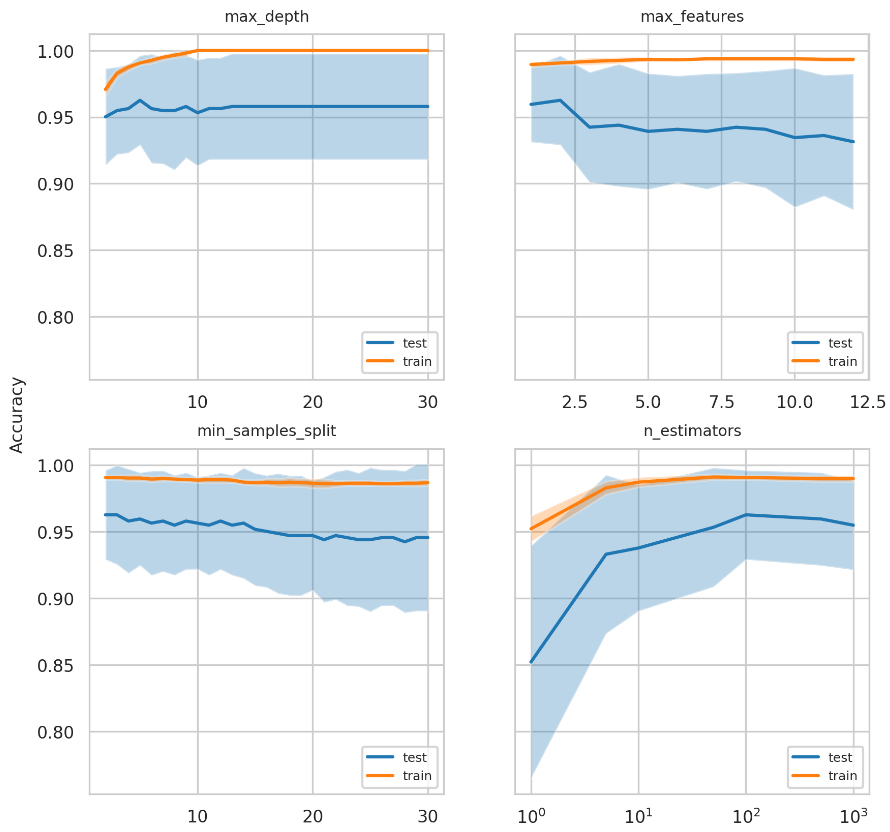


Figure 1. Parameters selection (step 2): Dependancy of accuracy at test (blue) and train (orange) parts of the dataset on the parameter value is shown. The mean+/-standard deviation of 5 folds is shown.

Best parameters 1:{'max_depth': 5, 'max_features': 2, 'min_samples_split': 2, 'n_estimators': 100}

Best parameters 2:{'max_depth': 5, 'max_features': 2, 'min_samples_split': 2, 'n_estimators': 100}

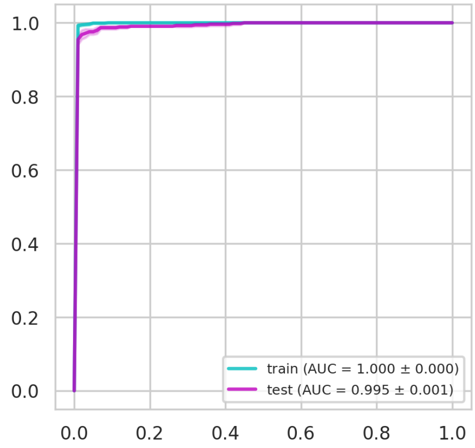


Figure 2. ROC curve at test (magenta) and train (blue) parts of the dataset is shown. The mean+/-standard deviation of 5 folds is shown.

OOB estimations: {'oob_accuracy': '0.972 ± 0.003', 'oob_f1_score': '0.972 ± 0.003', 'oob_precision': '0.982 ± 0.004', 'oob_recall': '0.963 ± 0.004'}

Model: Genus_MW-Holm

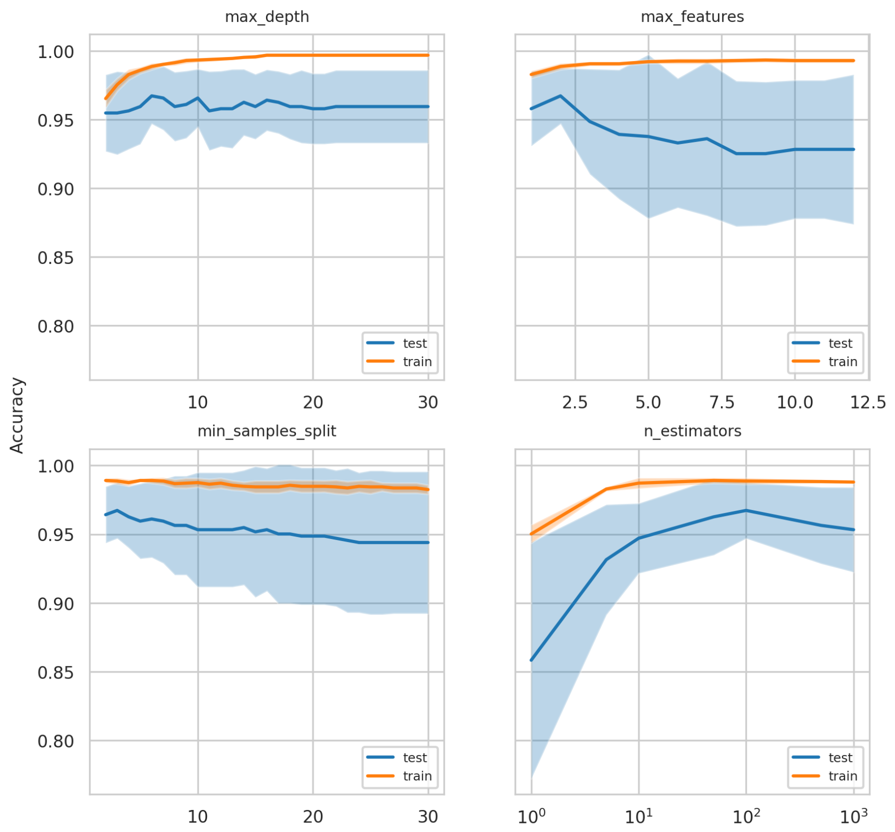


Figure 1. Parameters selection (step 2): Dependancy of accuracy at test (blue) and train (orange) parts of the dataset on the parameter value is shown. The mean+/-standard deviation of 5 folds is shown.

Best parameters 1:{'max_depth': 6, 'max_features': 2, 'min_samples_split': 3, 'n_estimators': 100}

Best parameters 2:{'max_depth': 6, 'max_features': 2, 'min_samples_split': 3, 'n_estimators': 100}

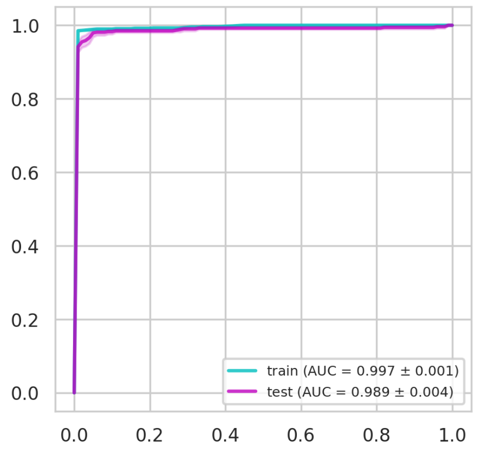


Figure 2. ROC curve at test (magenta) and train (blue) parts of the dataset is shown. The mean+/-standard deviation of 5 folds is shown.

OOB estimations: {'oob_accuracy': '0.967 ± 0.003', 'oob_f1_score': '0.967 ± 0.003', 'oob_precision': '0.981 ± 0.004', 'oob_recall': '0.953 ± 0.005'}
